# Physicochemical Properties and Whey Proteomes of Camel Milk Powders Produced by Different Concentration and Dehydration Processes

**DOI:** 10.3390/foods11050727

**Published:** 2022-03-01

**Authors:** Zhengzheng Zou, John A. Duley, David M. Cowley, Sarah Reed, Buddhika J. Arachchige, Bhesh Bhandari, Paul N. Shaw, Nidhi Bansal

**Affiliations:** 1 School of Agriculture and Food Sciences, The University of Queensland, St. Lucia, QLD 4072, Australia; zhengzheng.zou@uq.edu.au (Z.Z.); b.bhandari@uq.edu.au (B.B.); 2 School of Pharmacy, The University of Queensland, Woolloongabba, QLD 4102, Australia; johnaduley@gmail.com (J.A.D.); n.shaw@uq.edu.au (P.N.S.); 3 Mater Research Institute, The University of Queensland, Woolloongabba, QLD 4102, Australia; dcowley@bigpond.net.au; 4 Centre for Clinical Research, The University of Queensland, Herston, QLD 4006, Australia; sj.reed@uq.edu.au (S.R.); b.jayakody@uq.edu.au (B.J.A.)

**Keywords:** milk concentration, milk drying, camel milk powder, whey proteome, bioactive proteins

## Abstract

Camel milk powder production is an alternative to preserve the perishable milk for later-date consumption. However, the impacts of dehydration processes on bioactive compounds in camel milk are largely unknown. Hence, the present study attempted to compare the physicochemical properties and protein profiles of camel milk powders produced by different concentration and dehydration processes. Six camel milk powders were produced by freeze- and spray-drying methods in conjunction with two liquid concentration techniques, namely spray dewatering and reverse osmosis. The results of proteomic analysis showed that direct freeze-dried camel milk powder had the least changes in protein profile, followed by direct spray-dried powder. The camel milk powders that underwent concentration processes had more profound changes in their protein profiles. Among the bioactive proteins identified, lactotransferrin and oxidase/peroxidase had the most significant decreases in concentration following processing. On the contrary, glycosylation-dependent cell adhesion molecule 1, peptidoglycan recognition protein 1, and osteopontin increased in concentration. The results revealed that direct freeze drying was the most ideal method for preserving the bioactive proteins during camel milk powder production. However, the freeze-drying technique has cost and scalability constraints, and the current spray-drying technique needs improvement to better retain the bioactivity of camel milk during powder processing.

## 1. Introduction

Camel milk has become topical in recent years because of its nutritional value [1]. As compared to bovine milk, camel milk is richer in vitamin C, essential minerals, and bioactive proteins, such as lactotransferrin (LTF), glycosylation-dependent cell adhesion molecule 1 (GLYCAM1), peptidoglycan recognition protein 1 (PGRP1), and whey acidic protein (WAP) [2]. Camel milk lacks beta-lactoglobulin, which is one of the major protein allergens in bovine milk [3]. Several studies have also shown evidence of camel milk having prospective therapeutic properties, such as antidiabetic and antiautism, which is associated with their abundant level of bioactive peptides/proteins [2]. Despite the high nutritional and potential medicinal value of camel milk, its global supply is limited because camels are typically raised in arid places such as deserts. Therefore, the production of dried powder from camel milk without impairing its bioactive components is highly desirable not only to make it available worldwide but also to extend its shelf life, reduce transportation costs, and expand its applications. Although camel milk powder is becoming an increasingly important dairy product, studies on camel milk powder production are still limited, and it is unclear how different processing conditions affect the inherent bioactivity of camel milk [4].

Most of the commercial camel milk powder products available in Australia are freeze-dried. The core principle of freeze drying is sublimation, which is the removal of water at a pressure well below the triple point of water. Milk is frozen prior to sublimation under high vacuum, leaving its solid components such as protein and carbohydrates behind. From a theoretical perspective, freeze drying would retain heat-liable bioactive components since no heat treatment is involved. However, proteins and enzymes can either undergo structural changes or aggregation, or even both, during freeze drying, and these developments can damage their biological activities. Chemical changes, such as deamidation of asparagine residues and hydrolysis of peptide bonds (next to aspartic acid residues), have been reported to occur during freeze drying, leading to destabilisation of the proteins [5]. Key disadvantages of freeze drying include high running costs and a long processing time, resulting in high retailing prices for freeze-dried camel milk powder and limiting its application to industrial-scale production for economical dairy products.

Spray drying, on the other hand, is the most widely used dehydration technique for dairy powder production. An atomiser or spray nozzle is used to disperse the liquid into a controlled drop-size spray, which is subsequently dried as it passes through a flow of hot gas. Spray drying is an efficient and economical drying approach for large-scale processing. However, the heat treatment may be detrimental to temperature-sensitive components in the milk. It is known that excessive heat during processing causes free -SH groups to interact with the S-S bond of cysteine-containing proteins. As a result, these -SH/S-S-interchange reactions lead to the irreversible aggregation of proteins into protein complexes [6]. Hydrophobic interactions and hydrogen bonding have also been reported to play a crucial role in heat-induced milk protein association [7]. Several studies have attempted to determine the optimal spray-drying conditions for camel milk powder production. However, most of these studies focused on evaluating the optical, surface, or physicochemical properties of camel milk powders [8,9]. Recently, changes to major proteins in spray-dried dromedary camel milk were investigated by Zouari et al. [10] using liquid chromatography–mass spectrometry (LC-MS). The concentrations of camel milk PGRP and caseins remained stable, while serum albumin (SA) and α-lactalbumin (α-LA) decreased after drying. In another study by Li et al. [11], protein profiles of unprocessed Bactrian camel milk were compared with camel milk powder (dried using heat). Approximately 20% of proteins quantified in Bactrian camel milk powder were found to have undergone profound changes as compared with unprocessed camel milk. Comprehensive analysis on the changes in bioactive proteins and bioactivities in dromedary camel milk powder is still lacking presently.

In industrial-scale production of bovine milk powder, ‘falling-film evaporation’ is a common method of milk concentration prior to spray drying. In addition to thermal evaporation methods, reverse osmosis (RO) has also been used for milk concentration, as the relatively low processing temperature minimises thermal destruction of thermosensitive bioactive components [12]. Milk concentration reduces the energy required for subsequent spray drying [13]. Moreover, longer shelf life, larger powder particles, and better rehydration properties have been observed for milk powders produced from concentrated milk, suggesting the concentration step has a positive influence on the physical properties of the dried milk powder [14]. The higher total solid content in bovine milk has also been reported to contribute to the preservation of whey proteins during drying [15]. However, there is limited information on the influences of concentration processes on physicochemical properties and bioactivities of camel milk powders.

The objective of the present study is to compare the physicochemical properties and protein profiles of camel milk powders produced by different concentration and dehydration processes. Results from this study can provide valuable information and contribute to the current understanding of camel milk powder production.

## 2. Materials and Methods

### 2.1. Materials

Camel milk samples were collected from a local camel farm (QLD, Australia). The milk samples were kept at 4 °C and processed within 48 h of collection. Each treatment was performed in triplicate. Formic acid, Pierce™ BCA Protein Assay Kit, and the SOLAμ HRP 96-well plate were purchased from Thermo Fisher Scientific Pty. Ltd. (Scoresby, VIC, Australia). Trypsin Gold was purchased from Promega Australia (Sydney, NSW, Australia). All the other chemicals were obtained from Sigma-Aldrich Pty. Ltd. (Castle Hill, NSW, Australia) unless otherwise noted. Water was high-grade deionised (>18.5 megaOhm) water.

### 2.2. Concentration of Camel Milk by Spray Dewatering

Camel milk was concentrated using spray-dewatering (SD) equipment designed at the University of Queensland. However, details of the equipment cannot be disclosed, as a patent application is being considered. The product temperature during concentration was maintained at 35 °C. To avoid protein denaturation during a lengthy process, SD concentration was stopped when the total solids content of camel milk attained 20% *w*/*w*.

### 2.3. Concentration of Camel Milk by Reverse Osmosis

Reverse osmosis (RO) was conducted as a batch process. The milk was circulated between a small bulk tank and the filtration plant until the volume was reduced by half. The 1812 spiral-wound membrane used for RO was produced by Guochu Technology Co. Ltd. (Xiamen, China). Pressure across the RO membrane was set at 30 bar, and the temperature was kept below 50 °C by circulating cooling water during processing. To avoid thermal inactivation and interfacial inactivation (caused by milk foaming during the RO process) of bioactive proteins, RO concentration was stopped when the total solids content of camel milk reached 20% *w*/*w*.

### 2.4. Dehydration by Spray Drying of Camel Milk

Spray drying was undertaken on raw milk and both types of concentrated milk (SD and RO) using a concurrent Anhydro spray dryer (the University of Queensland, St. Lucia, Australia), which was equipped with a twin fluid nozzle and had a water evaporation capacity of about 4 L/h. The compressed air at the inlet of the atomiser was set at 40 kPa. The inlet and outlet temperatures of the drying air were controlled at 160 and 70 °C, respectively. The powders were collected from the cyclone separator and stored in vacuum-sealed aluminium-coated plastic pouches at −20 °C for further analyses.

### 2.5. Dehydration by Freeze Drying of Camel Milk

Camel milk (raw, or concentrated by SD or RO) was frozen at −20 °C for two days, then dried using a vacuum freeze dryer (John Morris Scientific Pty. Ltd., Murarrie, QLD, Australia). The condenser temperature was maintained at approx. −55 °C, and the vacuum was approx. 0.2 mbar. The powders were stored in vacuum-sealed aluminium-coated plastic pouches at −20 °C for further analyses.

### 2.6. Physicochemical Properties of Camel Milk Powders

Colour, true density, water activity, moisture content and solubility analyses, X-ray diffraction analysis (XRD), and scanning electron microscopy (SEM) of the camel milk powders produced were performed according to Ho et al. [16].

### 2.7. Lactoperoxidase Activity of Camel Milk Powder

Camel milk powder was reconstituted in Milli-Q water equivalent to the total solids content of corresponding raw milk. The lactoperoxidase (LPO) activity was assayed using the fluorescent AR^®^/Resorufin method [17].

### 2.8. Proteomics Comparison of Raw Milk and Camel Milk Powders

Aqueous solutions of camel milk powders were prepared by adding the powders into Milli-Q water with constant stirring, equivalent to the total solids content corresponding to raw milk. The reconstituted camel milk, together with camel raw milk, was ultracentrifuged at 100,000× *g* for 60 min at 25 °C (Beckman JXN-30 ultracentrifuge, with rotor JA-30.50, Beckman, Brea, CA, USA). The middle layer of milk serum was used for protein quantification using Pierce™ BCA Protein Assay Kit. The milk serum obtained through ultracentrifugation was diluted in Milli-Q water to get a 1 mg/mL protein solution.

For proteomic analysis, protein digestion was directly conducted on filter membrane of a Pall Nanosep with 10 K omega filter (PALL Corporation, Washington, NY, USA) by mixing 100 μL diluted milk serum with 100 μL 50 mM ABC (ammonium bicarbonate in water, pH 8) on the membrane. The fractions were reduced with 1 mM dithiothreitol, alkylated with 2.5 mM iodoacetamide, washed with 100 μL 50 mM ABC three times, and digested with 1 μg trypsin (37 °C, overnight). After overnight trypsin digestion, 100 μL 0.1% formic acid was added to the membrane, and the sample was centrifuged at 15,000× *g* for 5 min. The eluate was mixed with 200 μL 0.1% trifluoroacetic acid and desalted using the SOLAμ HRP 96-well plate following the manufacturer’s instruction. The eluted peptides were dried in a vacuum centrifuge and resuspended in 0.1% formic acid for HPLC-MS analysis.

The information-dependent acquisition was carried out on an AB SCIEX TripleTOF 5600 mass spectrometer (ABSCIEX, Redwood City, CA, USA) coupled to a Nano Ultra 1D + HPLC system (Eksigent, Redwood City, CA, USA) as described by Jayabalan et al. [18]. Full-scan (survey scan) mass spectra were acquired for 0.2 s in high sensitivity mode from 300 to 1700 m/z, followed by collision-induced dissociation data-dependent product ion scan (MS/MS) of the most abundant ions (Top 25) from a survey scan. Identical LC-MS conditions were used for SWATH-MS on the same instrument. Using an isolation width of 26 Da (containing 1 Da for the window overlap), a set of 32 overlapping windows was constructed covering the precursor mass range of 400–1200 Da for SWATH acquisition. An accumulation time of 0.08 s was used for all fragment ion scans, and the high sensitivity mode was selected.

Product ion spectra were searched using the Paragon search engine on ProteinPilot™ Software (Version 5.0, AB Sciex, Redwood City, CA, USA). The Camelus (Taxon identifier 9836) reference database was downloaded as fasta files from UniProt (http://www.uniprot.org/, accessed on 1 July 2021). The results from ProteinPilot were used as an ion library to measure the abundance of peptides and proteins using PeakView 2.1 (SCIEX) with the following settings: shared peptides, excluded; peptide confidence threshold, 99%; false discovery rate (FDR), 1%; extracted-ion chromatogram extraction window, 6 min; extracted-ion chromatogram width, 75 ppm. For protein-centric analyses, protein abundances were normalised to total protein in a sample. Peakview output was reformatted with a Python script to eliminate low-quality data by applying a peptide FDR cut-off of 1%, and protein abundance differences between samples were determined using MSstats (2.4) in R as described by Kerr et al. [19]. Principal component analysis (PCA) was performed using Python, the machine learning library Scikit-learn (0.19.1), and the data visualisation package Plotly (1.12.2).

## 3. Results and Discussion

### 3.1. Concentration and Dehydration of Camel Milk

The concentration of camel milk was performed by SD, which removes water as a vapour using hot air, or by RO, which removes water through membrane filtration. On the basis of total solids content, the concentration factors of both SD and RO processes were between 1.6 and 1.7 (Table S1). The raw and concentrated camel milk samples were either freeze dried or spray dried subsequently.

The samples used for this investigation included raw milk (RM) and six different camel milk powders. A breakdown of the samples prepared by different processing techniques is as follows:

Six camel milk powders:
Freeze-dried raw milk powder (FR);
SD-concentrated/freeze-dried milk powder (FSD);
RO-concentrated/freeze-dried milk powder (FRO);
Spray-dried raw milk powder (SR);
SD-concentrated/spray-dried milk powder (SSD);
RO-concentrated/spray-dried milk powder (SRO).

During spray drying, only milk powder in the product collection chamber below the cyclone separator was retrieved for subsequent analysis. Milk powder stuck onto the drying chamber was discarded to avoid the incorporation of outliers that are potentially overheated. In addition, a proportion of the milk powder was lost in the exhaust gas from the cyclone separator, resulting in a low yield (24.7–34.0%, Table S2). However, it appeared that concentration step and type of concentration did not have any effect on powder yield. Freeze drying, on the other hand, recovered more than 95% of the dry matter in the milk samples.

### 3.2. Physicochemical Properties of the Camel Milk Powders

Water activity, moisture content, colour, true density, and solubility of camel milk powders produced from the different combinations of concentration and dehydration are summarised in Table 1. In general, the freeze-dried camel milk powders had lower water activity and moisture content as compared with spray-dried camel milk powders. Lower water activity in a powder is usually associated with greater stability and longer shelf life of the product [20]. Schuck et al. suggested that water activity of milk powders should be close to 0.2 for optimal preservation [21]. Lower moisture content was also reported for freeze-dried coconut milk powder than spray-dried [22]. Spray-dried goat milk powders showed similar water activity (0.239–0.259) to spray-dried camel milk powders in the present study [23]. The spray-dried camel milk powders were significantly whiter than freeze-dried camel milk powders (Table 1).

True densities of the spray-dried camel milk powders were similar to values reported by Ho et al. [16] but were significantly lower than those of freeze-dried camel milk powders (Table 1). An increased moisture content usually decreases the true density of an amorphous powder [24]. Therefore, the lower true density of spray-dried camel milk powder could be ascribed to their higher moisture content. The lower true densities of the spray-dried camel milk powders may be an indication of a greater amount of intraparticle pores that might have been generated from thermal devolatilisation of milk carbohydrates but could not be penetrated by nitrogen gas during measurement [25]. Although the amorphous or crystalline state of powder has also been reported to affect powder true density [16], no differences in amorphous/crystalline structure were observed between freeze-dried and spray-dried camel milk powders, as discussed in the following. Freshly freeze-dried and spray-dried camel milk powders dissolved well in water. Ho et al. also reported good solubility (98.62%) for freshly spray-dried camel milk powder, and the solubility level remained stable for at least 9 weeks of storage based on their results [26].

**Figure 1:**
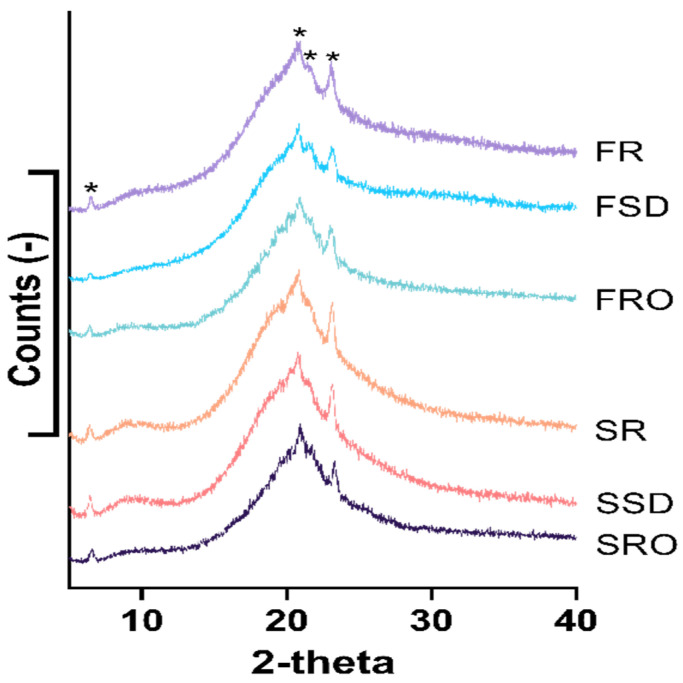
X-ray diffraction (XRD) analysis of camel milk powders. FR, freeze-dried raw milk powder; FSD, spray-dewatering-concentrated/freeze-dried milk powder; FRO, reverse-osmosis-concentrated/freeze-dried milk powder; SR, spray-dried raw milk powder; SSD, spray-dewatering-concentrated/spray-dried milk powder; SRO, reverse-osmosis-concentrated/spray-dried milk powder. *, Four crystal forms at diffraction angles (2θ) 6.4, 20.8, 21.6, and 23.1, respectively, were identified in the camel milk powders.

The XRD spectra in Figure 1 show the diffractogram of the scattering patterns, which provide insights into the crystalline or amorphous structure of the camel milk powders. The intensities and diffraction patterns of X-rays were quite similar for all the six camel milk powder samples, indicating that different concentration and dehydration techniques did not have a notable influence on the amorphous/crystal structure in the final camel milk powder product. A single large peak with some smaller sharp peaks was observed on the XRD scattering patterns, suggesting that the predominant fraction of camel milk powder existed as an amorphous structure with some amount of crystalline. Four crystal forms (marked with “*” in Figure 1) at diffraction angles (2θ) 6.4, 20.8, 21.6, and 23.1, respectively, were identified in the camel milk powders. These four sharp peaks are attributed to the diffractions of small amounts of lactose and milk fat crystals that were formed during the processing and storage of camel milk powders [27,28,29].

**Figure 2:**
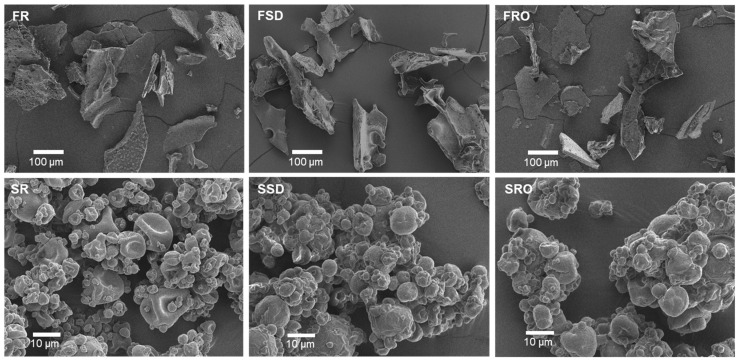
Scanning electron microscopy (SEM) images of camel milk powders. FR, freeze-dried raw milk powder; FSD, spray-dewatering-concentrated/freeze-dried milk powder; FRO, reverse-osmosis-concentrated/freeze-dried milk powder; SR, spray-dried raw milk powder; SSD, spray-dewatering-concentrated/spray-dried milk powder; SRO, reverse-osmosis-concentrated/spray-dried milk powder.

The SEM images of freeze-dried and spray-dried camel milk powders are shown in Figure 2. The former have irregular structures, while the latter are characterised by spherical-shaped particles with wrinkled and folded surfaces. These images are similar to those reported previously for bovine and camel milk powders [30,31]. Compared with the powder particles from SR shown here, more agglomerated particles were observed for SSD and SRO (Figure 2). In agreement with our findings, Balde and Aïder [32] reported that cryo-concentrated and spray-dried bovine milk powder had predominantly agglomerated particles. High lactose content has been reported to generate more wrinkled particles [33]. However, the differences between surface morphology of camel milk powders with or without concentration were not notable, perhaps due to the relatively low concentration factors in this study.

### 3.3. Quantitative Proteomics of Whey Protein Changes Following Camel Milk Powder Processing

#### 3.3.1. Identification of Whey Proteins before and after Processing

**Figure 3:**
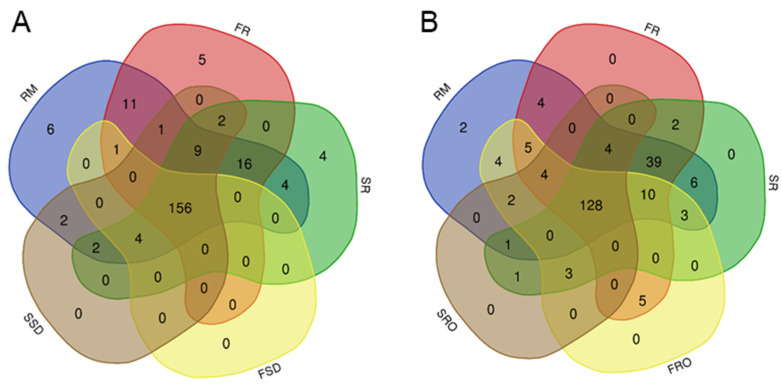
Venn diagrams of camel serum proteins identified in the spray-dewatering (SD) group (**A**) and reverse osmosis (RO) group (**B**). RM, raw milk; FR, freeze-dried raw milk powder; SR, spray-dried raw milk powder; FSD, spray-dewatering-concentrated/freeze-dried milk powder; SSD, spray-dewatering-concentrated/spray-dried milk powder; FRO, reverse-osmosis-concentrated/freeze-dried milk powder; SRO, reverse-osmosis-concentrated/spray-dried milk powder.

Venn diagram analyses of the quantified proteins from the SD and RO milk concentration groups are shown in Figure 3. There were 212, 201, 197, 161, 176, 164, and 143 proteins identified in RM, FR, SR, FSD, SSD, FRO, and SRO, respectively. Among the 223 proteins identified in raw milk and six milk powders, 156 and 128 proteins were present across SD- and RO-group powders, respectively. By comparison to the 212 proteins identified in raw milk, there were 18, 21, 51, 38, 56, and 73 proteins missing from FR, SR, FSD, SSD, FRO, and SRO, respectively. This indicates that direct drying of camel milk without any preconcentration step had the least influence on protein components of the final product. The concentration processes, either SD or RO, prior to camel milk drying, markedly altered the protein components of the camel milk powder produced, with RO having a greater effect than SD.

α-Actinin-4 isoform 1, antigen p97 melanoma-associated protein, 6-phosphogluconate dehydrogenase, mesothelin, neutrophilic granule protein-like, and elongation factor 2 were absent in FR, FSD, or FRO. This revealed that these proteins might have been obliterated after freeze drying. According to Gene Ontology (GO) annotation, they are involved in actin/calcium ion binding (α-actinin-4 isoform 1), ATP/microtubule/NADP binding (6-phosphogluconate dehydrogenase), GTP binding (elongation factor 2), catalytic activity (6-phosphogluconate dehydrogenase and elongation factor 2) and defence response (neutrophilic granule protein-like).

A total of 12 proteins present in RM were not detected in any milk powders processed by spray drying (SR, SSD, and SRO). Over half of them have also been annotated to have binding function, including calcium binding (calcium-binding protein and calmodulin), ATP/unfolded protein binding (endoplasmin), cobalamin binding (transcobalamin-2), RNA binding (uncharacterised protein), DNA binding (histone H1t), and Guanine nucleotide binding (Guanine nucleotide-binding protein).

#### 3.3.2. Quantification of Whey Proteins before and after Processing

**Figure 4:**
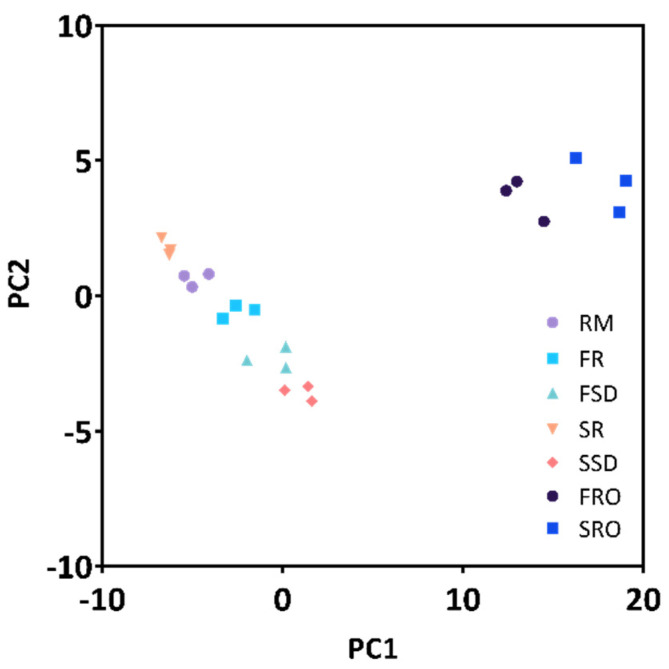
Principal component analysis of proteome of raw camel milk and processed camel milk powders. RM, raw milk; FR, freeze-dried raw milk powder; FSD, spray-dewatering-concentrated/freeze-dried milk powder; SR, spray-dried raw milk powder; SSD, spray-dewatering-concentrated/spray-dried milk powder; FRO, reverse-osmosis-concentrated/freeze-dried milk powder; SRO, reverse-osmosis-concentrated/spray-dried milk powder.

PCA was used to provide an overview of the proteomic variability between raw camel milk and the six camel milk powders (Figure 4). Clustering of the triplicate analyses of each milk/milk powder sample was observed. Among all the milk powders, FR and SR showed the closest association with RM. The milk powders that involved concentrating by RO (FRO and SRO) had clusters that were distinctively distant from the rest of the milk powders, indicating that their proteomes were more altered.

**Figure 5:**
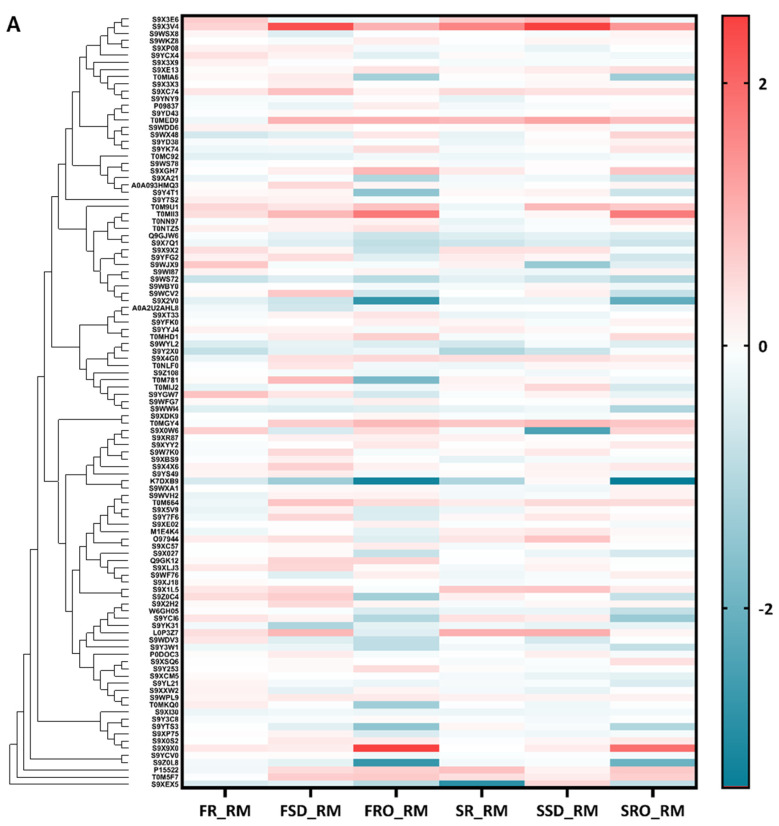

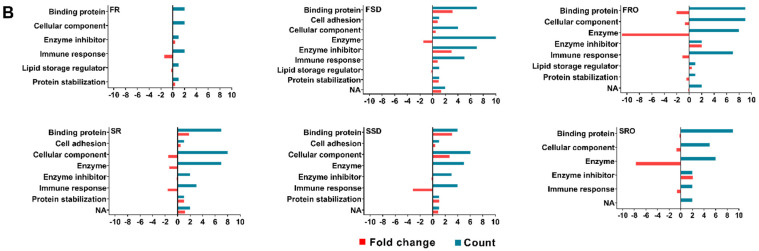
Heat map of abundance change of the proteins quantified in all the samples (**A**) and annotation of significantly changed proteins (**B**). Values shown as log2 (fold change) for proteins. RM, raw milk; FR, freeze-dried raw milk powder; FSD, spray-dewatering-concentrated/freeze-dried milk powder; FRO, reverse-osmosis-concentrated/freeze-dried milk powder; SR, spray-dried raw milk powder; SSD, spray-dewatering-concentrated/spray-dried milk powder; SRO, reverse-osmosis-concentrated/spray-dried milk powder.

For the six camel milk powders, a total of 108 proteins were successfully quantified for abundance as fold change in comparison to raw camel milk (Figure 5A). Consistent with the PCA analysis results, there was less change in protein abundance in FR and SR compared with RM, while the greatest fold change of protein abundance was marked in the RO-derived (FRO and SRO) powders. Among the 108 proteins quantified in all the samples, changes in the abundance of 9, 38, 39, 31, 25, and 26 proteins were significant (*p* < 10^−5^) in FR, FSD, FRO, SR, SSD, and SRO, respectively.

The proteins showing significant changes after processing were then grouped, based on their function annotation, in Figure 5B. Two proteins related to immune response (α-1-acid glycoprotein and platelet glycoprotein 4) were significantly decreased after freeze drying (FR). For FSD, most of the proteins with significant fold change were enriched after processing, except for some of the enzymes and perilipin (lipid storage regulator). In the case of FRO, a reduction in most of the protein categories, enzymes, and binding proteins in particular was observed.

Spray drying caused an abundant decrease in cellular components, enzymes, and proteins involved in immune response, while binding proteins, osteopontin (OPN, cell adhesion), κ-casein (κ-CN, protein stabilisation), and two uncharacterised proteins were enriched after spray drying (SR, Figure 5B). There was a nominal decrease in the enzyme inhibitors in SR. Zhang et al. [34] compared the whey proteomes in raw camel milk with spray-dried milk powder and found that the amount of enzymes and proteins exerting immunity activities were also reduced after processing, while protease inhibitors were relatively stable during spray drying. Spray-dewatering concentration prior to spray drying (i.e., SSD) produced a positive effect on the preservation of binding proteins, cellular components, and enzymes compared to spray drying alone (SSD vs. SR, Figure 5B). However, a more significant loss of proteins related to immune response was observed in SSD.

Similar to the protein profile change in FRO, enzymes were severely reduced in SRO, while enzyme inhibitors were well preserved. Slight reductions in the amounts of binding proteins, cellular components, and proteins related to immune response were also observed in SRO. The complete information of proteins showing significant changes after processing in each powder sample is detailed in Table S2.

Denaturation of whey proteins during spray drying of skimmed camel milk has been shown to be associated with processing temperature, and processing at either lower inlet or outlet temperatures resulted in lower overall denaturation values [35]. Although limited information is available in regards to the impact of drying processes on camel milk proteomes, several studies employed proteomic approaches to examine the effect of heat treatment on camel milk proteins [36,37]. Benabdelkamel et al. [37] showed that enzymes were the major fraction (61%) of heat-treatment-affected proteins in camel milk, followed by binding proteins (20%), cell adhesion proteins (10%), and proteins involved in the immune response (5%). In addition, it was observed that some key proteins such as serum albumin (SA), apolipoprotein, and carboxypeptidase were reduced significantly after heat treatment. This observation concurred well with the present study, in which the abundances of these proteins in SR, SSD, and SRO were also decreased markedly as compared with raw milk (Table S2). However, due to the different heat processing conditions and whey protein separation methods employed in present and the earlier study by Benabdelkamel et al. [37], variations in the preservation of some whey proteins were also observed. For example, hemopexin, a plasma glycoprotein that acts as a binding protein for iron and exerts antioxidant activity, did not change significantly in SR and SSD but decreased significantly at 98 °C in their study.

#### 3.3.3. Changes in Bioactive Proteins following Camel Milk Powder Processing

**Figure 6:**
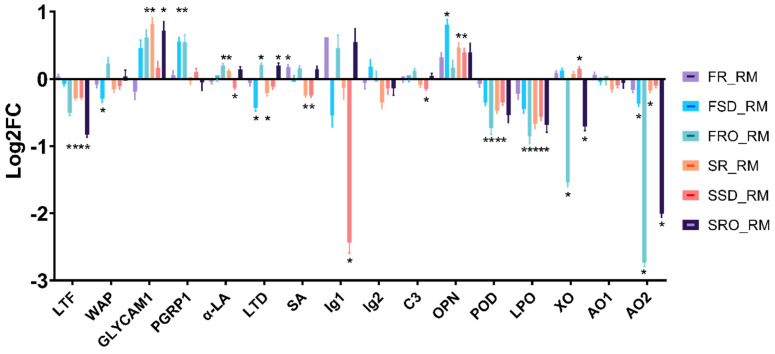
Fold change of bioactive proteins in the camel milk powders produced. LTF, Lactotransferrin (W6GH05); WAP, whey acidic protein (P09837); GLYCAM1, glycosylation-dependent cell adhesion molecule 1 (P15522); PGRP1, peptidoglycan recognition protein 1 (Q9GK12); α-LA, α-lactalbumin (S9YFK0); LTD, lactadherin (S9WF76); SA, serum albumin (S9WI87); Ig1, Ig-like domain-containing protein (S9X0W6); Ig2, Ig γ-3 chain C region (S9XBS9); C3, complement C3 (S9XDK9); OPN, osteopontin (S9XC74); POD, peroxidase (Q9GJW6); LPO, lactoperoxidase (S9X7Q1); XO, xanthine oxidase (S9Y4T1); AO1, amine oxidase (S9XJ18); AO2, amine aoxidase (S9Z0L8). UniProt accession numbers are given in brackets. RM, raw milk; FR, freeze-dried raw milk powder; FSD, spray-dewatering-concentrated/freeze-dried milk powder; FRO, reverse-osmosis-concentrated/freeze-dried milk powder; SR, spray-dried raw milk powder; SSD, spray-dewatering-concentrated/spray-dried milk powder; SRO, reverse-osmosis-concentrated/spray-dried milk powder. * indicates the change directly above or below it is significant (*p* < 10^−5^).

The abundance as fold change of bioactive proteins in the six camel milk powders is shown in Figure 6. LTF was observed to be unstable during the concentration and dehydration processing, with a significant amount of reduction in all of the camel milk powders, apart from FR and FSD. Other bioactive proteins showing significantly decreased concentrations in the camel milk powders included WAP in FSD, α-LA in SSD, lactadherin (LTD) in FSD and SR, SA in SR and SSD, Ig-like domain-containing protein (Ig1) in SSD, complement C3 (C3) in SSD, and peroxidase (POD) and LPO in all powders except for FR. A marked decrease in the concentration of xanthine oxidase (XO) and amine oxidase (AO) was observed in FRO and SRO, which may have resulted from the removal of a certain enzyme cofactor or ion during the RO processing.

GLYCAM1 was found to be enriched in all of the camel milk powders except for FR, although only the increases in FRO, SR, and SRO were identified as significant. OPN also seemed to be upregulated by the concentration and dehydration processing, with higher concentrations being observed in all powder samples. It is worth highlighting that both GLYCAM1 and OPN lack cysteine in their structures. During heat processing, irreversible aggregation of proteins into protein complexes is known to occur due to -SH/S-S interchange reactions, which involves the interaction of free -SH groups with the S-S bond of proteins [6,38]. Therefore, GLYCAM1 and OPN may not tend to aggregate during heating as both do not contain cysteine residues. Other bioactive proteins that were significantly enriched in the camel milk powders include PGRP1 (in FSD and FRO), α-LA (in FRO and SR), LTD (in FRO and SRO), SA (in FR), and XO (in SSD).

LTF is an iron-binding protein with several beneficial biological properties, such as antimicrobial, anticancer, and antioxidant activities [39]. Camel milk is known for its rich LTF content [40]. Downregulation of LTF by spray drying [34] or heat treatments [37,40] has been reported, suggesting the loss of LTF activity in thermally processed milk products. The C3, GLYCAM1, LTF, LTD, OPN, and XO in camel milk were reported to be greatly reduced from spray drying, with less than 10% protein retained in the powder produced, while 17% of α-LA and 35% of WAP were retained [34]; the prolonged heating process during spray drying may have led to the severe reduction in protein content observed in their study. It was recently reported by Zouari et al. [10] that 14.1%, 3.3%, and 0% of SA, α-LA, and PGRP, respectively, in camel milk were diminished after spray drying, which concurred well with the protein abundance changes reported in this study. During spray drying of acidic camel whey samples, PGRP also showed better stability than SA and α-LA [41]. Few other studies have investigated the effects of heat treatment on camel milk proteins. Similar to the results observed here, the degree of whey protein denaturation was found to be LTF > SA > α-LA > GLYCAM1 when camel milk was heated under varying conditions (75/85/90 °C, 5 min) [42]. α-LA was also found to be more heat stable than SA in camel milk by El-Agamy [40]. β-Lactoglobulin in bovine milk has been reported to interact with α-LA and promote aggregate formation during the heating process [6]. Therefore, the absence of β-lactoglobulin in camel milk might thus reinstitute the inherent heat stability of camel α-LA.

In bovine milk, SA, LTF, and αs2-CN were reported to form covalent complexes during heating [43]. Similar reactions might have also occurred in the present study when camel milk was heated during spray drying, leading to aggregation and denaturation of SA and LTF. Benabdelkamel et al. [37] reported that the order of heat denaturation followed LTF/LTD/SA > PGRP > α-LA/GLYCAM1 in camel milk. This trend is similar to that observed in the present study (Figure 6).

However, higher retainment of SA (62%), compared with PGRP (33%) and α-LA (0%) in camel milk after 80 °C heat treatment for 60 min has been reported [36]. Different protein extraction methods were used (urea incubation followed by filtration in the study conducted by Felfoul et al., as opposed to centrifugation extraction performed in other studies cited in the preceding) may account for the different residual protein amounts.

### 3.4. Lactoperoxidase Activities in Concentrated Camel Milk and Powders

Residual activities of the antimicrobial enzyme LPO after the different concentration and dehydration processes are shown in Table 2. For camel milk concentration, SD did not reduce the LPO activity significantly, while 37% of LPO activity was lost after RO. Among the six camel milk powders, FR and FSD retained significantly higher LPO activity with approximately 40–50% of activity remaining, while only approximately 10–20% of LPO activity remained in the other four powder samples. The observed changes in LPO activity were in agreement with the SWATH-MS quantification results of LPO (Figure 6).

The LPO activities in camel milk before and after SD were similar, indicating the mild heating in SD did not negatively affect the LPO activity. For RO, there is limited information on the changes in enzymatic activity during membrane concentration of milk. Syrios et al. [44] found that concentrating skim bovine milk by RO prior to drying altered the calcium concentration and pH of the retentate and its reconstituted powder. Changes in milk ionic environment and pH may consequently influence the LPO activities of the reverse-osmosis-concentrated milk (ROC) and RO powders. As whey is a severe fouling agent for RO membranes [45], it is possible that LPO may also be involved in deposit formation on membrane surfaces during RO processing, resulting in LPO activity loss in the RO retentate. Minerals, especially calcium phosphate, can also play an important role in fouling formation during RO, by acting as a binding bridge between the membrane and protein [45,46]. Thus, the high-affinity site for calcium in LPO may contribute to its binding to the RO membrane [47].

The LPO activity of raw camel milk decreased significantly after freeze drying (FR, Table 2). Although there is no heating involved in freeze drying, conformational changes and aggregation during freeze drying could result in protein inactivation [5]. The low moisture content of the freeze-dried camel milk powders (Table 1) may also have contributed to the loss of LPO activity. The residual moisture level was reported to be a critical variable affecting activity recovery of the freeze-dried enzyme, and a marked drop in activity recovery was observed at residual moisture levels less than 10% [48].

Severe heating during spray drying of raw and concentrated camel milk greatly reduced the LPO activity, resulting in much lower values observed in SR, SSD, and SRO. Thermal deactivation kinetics of camel milk LPO have been studied previously, and the half-time of camel milk LPO has been reported to be 4.45 min at 69 °C and 1.68 min at 71 °C [49]. On this basis, the enzyme has been identified as a promising marker to verify effective pasteurisation [50]. In the present study, LPO was also a good indicator to evaluate the possible loss of bioactive components caused by different processes due to its heat sensitivity and easy detection.

## 4. Conclusions

Six camel milk powders were produced from different combinations of concentration and dehydration processes. The physicochemical properties and changes in protein profiles of the camel milk powders were studied. Milk concentration did not have much influence on the physicochemical properties of the final milk powder produced but altered the protein profile significantly. LTF, POD, and LPO significantly decreased in all the spray-dried camel milk powders, while XO and AO2 decreased significantly in RO-processed powders (FRO and SRO). GLYCAM1, PGRP1, and OPN were the most stable bioactive whey proteins during milk processing. These results revealed that alternative large-scale techniques still need to be developed to preserve all the important bioactive proteins in camel milk, such as LTF. Future research might determine if further concentrating the milk benefits the preservation of bioactive components during the subsequent drying of camel milk.

## Figures and Tables

**Table 1:** Selected physicochemical properties of camel milk powders.

	FR	FSD	FRO	SR	SSD	SRO
Water activity	0.161 ± 0.057 ^a^	0.138 ± 0.020 ^a^	0.179 ± 0.039 ^a^	0.296 ± 0.050 ^b^	0.288 ± 0.031 ^b^	0.235 ± 0.021 ^ab^
Moisture, %	2.20 ± 0.19 ^ab^	1.82 ± 0.27 ^a^	2.74 ± 0.57 ^ab^	3.83 ± 1.22 ^b^	3.90 ± 0.70 ^b^	3.23 ± 0.20 ^ab^
Whiteness	89.8 ± 1.0 ^a^	88.6 ± 1.2 ^a^	87.4 ± 0.5 ^a^	94.4 ± 0.8 ^b^	94.8 ± 0.6 ^b^	95.2 ± 0.3 ^b^
True density, g/cm^3^	1.377 ± 0.007 ^a^	1.360 ± 0.059 ^a^	1.382 ± 0.001 ^a^	1.242 ± 0.022 ^b^	1.251 ± 0.017 ^b^	1.261 ± 0.009 ^b^
Solubility, %	98.99 ± 0.42 ^ab^	99.08 ± 0.29 ^a^	99.01 ± 0.24 ^ab^	98.24 ± 0.37 ^b^	98.33 ± 0.28 ^ab^	98.67 ± 0.17 ^ab^

FR, freeze-dried raw milk powder; FSD, spray-dewatering-concentrated/freeze-dried milk powder; FRO, reverse-osmosis-concentrated/freeze-dried milk powder; SR, spray-dried raw milk powder; SSD, spray-dewatering-concentrated/spray-dried milk powder; SRO, reverse-osmosis-concentrated/spray-dried milk powder. ^a^,^b^ Means with different superscripts within a row are significantly different (*p* < 0.05) from each other.

**Table 2:** Change of lactoperoxidase (LPO) activity during concentration and dehydration of camel milk.

Sample	Relative Activity (%)
RM	100.0 ± 0.9 ^a^
SDC	96.0 ± 5.6 ^a^
ROC	63.0 ± 6.3 ^b^
FR	49.8 ± 4.6 ^bc^
FSD	41.1 ± 3.7 ^c^
FRO	18.5 ± 2.3 ^d^
SR	13.9 ± 3.5 ^d^
SSD	8.5 ± 2.0 ^d^
SRO	9.0 ± 1.7 ^d^

RM, raw milk; SDC, spray-dewatering-concentrated milk; ROC, reverse-osmosis-concentrated milk; FR, freeze-dried raw milk powder; FSD, spray-dewatering-concentrated/freeze-dried milk powder; FRO, reverse-osmosis-concentrated/freeze-dried milk powder; SR, spray-dried raw milk powder; SSD, spray-dewatering-concentrated/spray-dried milk powder; SRO, reverse-osmosis-concentrated/spray-dried milk powder. ^a–d^ means the columns with different superscripts are significantly different (*p* < 0.05) from each other.

## Data Availability

The data presented in this study are available on request from the corresponding author.

## Supplementary Materials

The following supporting information can be downloaded at: https://www.mdpi.com/article/10.3390/foods11050727/s1, Table S1: Summary of concentration and dehydration processes of camel milk, Table S2: Proteins showing significant change after camel milk powder processing.
