# Supplementary material for: Physicochemical Properties and Whey Proteomes of Camel Milk Powders Produced by Different Concentration and Dehydration Processes
Source: Foods. 2022 Mar 1;11(5):727. doi: 10.3390/foods11050727 (PMC8909633; doi:10.3390/foods11050727)
Supplement: Supplementary file 1 [file foods-11-00727-s001.zip › foods-1593985-supplementary.pdf]

Supplementary Materials

Table S1. Summary of concentration and dehydration processes of camel milk.

Table S2. Proteins showing significant change after camel milk powder processing.

Table S1. Summary of concentration and dehydration processes of camel milk.

**Process**	**Concentration factor**	**Drying yield (%)**
Spray drying		
Raw milk - Batch 1		32.4
Raw milk - Batch 2		26.1
Raw milk - Batch 3		24.7
Concentrated milk (SD) - Batch 1	1.6	34.0
Concentrated milk (SD) - Batch 2	1.6	30.7
Concentrated milk (SD) - Batch 3	1.7	28.4
Concentrated milk (RO) - Batch 1	1.7	25.3
Concentrated milk (RO) - Batch 2	1.6	32.9
Concentrated milk (RO) - Batch 3	1.6	26.1
Freeze drying		
Raw milk - Batch 1		98.2
Raw milk - Batch 2		97.9
Raw milk - Batch 3		96.9
Concentrated milk (SD) - Batch 1	1.6	96.4
Concentrated milk (SD) - Batch 2	1.6	97.1
Concentrated milk (SD) - Batch 3	1.7	97.9
Concentrated milk (RO) - Batch 1	1.7	96.2
Concentrated milk (RO) - Batch 2	1.6	98.7
Concentrated milk (RO) - Batch 3	1.6	98.4

SD, spray dewatering; RO, reverse osmosis.

Table S2. Proteins showing significant change after camel milk powder processing.

**Sample**	**Accession**	**Protein name**	**Function of protein**	**Relative amount to raw milk**	**p value**
FR	L0P3Z7	κ-Casein	Protein stabilization	134 ±3%	0
	T0MII3	α-2-Macroglobulin-like protein	Enzyme inhibitor	134 ±3%	0
	S9WDD6	CLb domain-containing protein	Binding protein	113 ±2%	3.03E-06
	S9WI87	Serum albumin	Cellular component	113 ±2%	1.27E-07
	M1E4K4	β-Casein	Cellular component	85 ±1%	0
	S9YK74	Perilipin	Lipid storage regulator	84 ±3%	1.25E-07
	S9XI30	FABP domain-containing protein	Binding protein	83 ±4%	6.71E-06
	S9WX48	α-1-Acid glycoprotein	Immune response	66 ±6%	6.86E-11
	S9Y2X0	Platelet glycoprotein 4	Immune response	57 ±5%	0
FSD	S9X3V4	TAF domain-containing protein	Binding protein	495 ±12%	0
	T0MED9	Uncharacterized protein	Cellular component	202 ±6%	0
	T0MII3	α-2-Macroglobulin-like protein	Enzyme inhibitor	191 ±3%	0
	L0P3Z7	κ-Casein	Protein stabilization	191 ±3%	0
	T0M781	α-2-Antiplasmin isoform 2	Enzyme inhibitor	187 ±14%	5.742E-06
	S9XC74	Osteopontin	Cell adhesion	175 ±6%	0
	T0M664	Zinc-α-2-glycoprotein-like protein	Binding protein	168 ±4%	0
	S9WCV2	Sulfhydryl oxidase	Enzyme	164 ±8%	1.084E-09
	T0M5F7	Uncharacterized protein	NA	158 ±7%	6.098E-11
	T0MGY4	Uncharacterized protein	NA	157 ±9%	1.307E-06
	S9X4X6	Uncharacterized protein	Immune response	151 ±4%	0
	Q9GK12	Peptidoglycan recognition protein 1	Immune response	147 ±5%	0
	S9Y7F6	Cystatin domain-containing protein	Enzyme inhibitor	143 ±8%	6.597E-06
	A0A093HMQ3	Growth/differentiation factor 8	Binding protein	141 ±5%	3.002E-13
	S9X1L5	Lipoprotein lipase isoform 3	Enzyme	141 ±7%	1.307E-06
	S9W7K0	Uncharacterized protein	Immune response	140 ±5%	2.543E-13
	S9X2H2	Uncharacterized protein	Enzyme inhibitor	139 ±3%	0
	S9YFG2	Complement factor D	Enzyme	136 ±7%	4.506E-06
	O97944	α_{s2}-Casein	Cellular component	133 ±4%	5.195E-12

	S9X4G0	Neutrophil gelatinase-associated lipocalin-like protein	Binding protein	133 ±4%	3.376E-12
	P15522-2	Glycosylation-dependent cell adhesion molecule 1	Cellular component	129 ±4%	2.71E-10
	S9WPL9	Uncharacterized protein	Enzyme inhibitor	121 ±2%	6.661E-15
	S9X0S2	Uncharacterized protein	Enzyme	121 ±4%	5.057E-06
	T0MIA6	Gelsolin isoform 32	Binding protein	118 ±3%	3.507E-06
	T0NN97	Uncharacterized protein	Enzyme inhibitor	113 ±2%	1.718E-07
	S9YK74	Perilipin	Lipid storage regulator	86 ±3%	2.069E-06
	P09837	Whey acidic protein	Enzyme inhibitor	82 ±4%	7.813E-07
	Q9GJW6	Peroxidase	Enzyme	79 ±3%	3.035E-12
	S9Z0L8	Amine oxidase	Enzyme	78 ±3%	0
	S9YTS3	Actin, cytoplasmic 1	Binding protein	76 ±3%	0
	S9WX48	α-1-Acid glycoprotein	Immune response	75 ±6%	4.174E-06
	S9WF76	Lactadherin	Immune response	74 ±3%	0
	S9X7Q1	Lactoperoxidase	Enzyme	73 ±5%	9.851E-10
	S9WSX8	Legumain	Enzyme	73 ±4%	2.189E-11
	A0A2U2AHL8	Ribonuclease HII	Binding protein	65 ±4%	1.254E-13
	S9X2V0	UDP-Gal:βGlcNAc β 1,4-galactosyltransferase 1, membrane-bound form-like protein	Enzyme	61 ±7%	3.035E-12
	S9YK31	Peptidyl-prolyl cis-trans isomerase	Enzyme	46 ±11%	5.884E-12
	K7DXB9	α_{s1}-Casein	Cellular component	40 ±13%	7.782E-13
FRO	S9X9X0	Vitelline membrane outer layer protein 1-like protein	Cellular component	563 ±15%	0
	T0MII3	α-2-Macroglobulin-like protein	Enzyme inhibitor	333 ±4%	0
	T0MED9	Uncharacterized protein	Cellular component	201 ±9%	2.273E-14
	S9X3V4	TAF domain-containing protein	Binding protein	200 ±12%	2.836E-07
	S9XGH7	Polyubiquitin-B	Binding protein	192 ±7%	5.978E-12
	T0MGY4	Uncharacterized protein	NA	188 ±7%	4.293E-15
	T0M9U1	Hermansky-Pudlak syndrome 5 protein homolog	Cellular component	174 ±11%	6.723E-06
	P15522	Glycosylation-dependent cell adhesion molecule 1	Cellular component	153 ±9%	1.79E-06
	T0MHD1	MHC_I-like_Ag-recog domain-containing protein	Immune response	153 ±7%	1.694E-07
	Q9GK12	Peptidoglycan recognition protein 1	Immune response	146 ±8%	1.79E-06

S9X4G0	Neutrophil gelatinase-associated lipocalin-like protein	Binding protein	142 ±2%	0
S9YK74	Perilipin	Lipid storage regulator	134 ±5%	2.836E-07
S9XYY2	Hemopexin	Binding protein	123 ±3%	2.774E-10
S9WPL9	Uncharacterized protein	Enzyme inhibitor	121 ±3%	1.62E-07
S9WF76	Lactadherin	Immune response	115 ±2%	1.378E-11
S9YFK0	α-Lactalbumin	Enzyme	115 ±2%	1.479E-08
S9W7K0	Uncharacterized protein	Immune response	91 ±2%	6.864E-07
S9XI30	FABP domain-containing protein	Binding protein	83 ±3%	1.496E-07
M1E4K4	β-Casein	Cellular component	78 ±3%	1.592E-13
L0P3Z7	κ-Casein	Protein stabilization	73 ±6%	2.094E-06
O97944	α_{s2}-Casein	Cellular component	73 ±5%	1.552E-10
W6GH05	Lactotransferrin	Immune response	70 ±2%	0
S9XP75	Monocyte differentiation antigen CD14	Immune response	70 ±4%	0
S9YGW7	Heparin cofactor 2	Cellular component	66 ±8%	1.74E-06
Q9GJW6	Peroxidase	Enzyme	60 ±7%	1.935E-11
S9X7Q1	Lactoperoxidase	Enzyme	55 ±8%	6.288E-12
S9WDV3	Fibrinogen γ chain isoform γ-B	Binding protein	54 ±6%	0
S9Y3W1	Glucose-regulated protein	Binding protein	54 ±11%	1.651E-07
S9XEX5	Myelin proteolipid protein isoform 2	Cellular component	51 ±5%	0
S9YCI6	Peptidyl-prolyl cis-trans isomerase	Enzyme	48 ±11%	1.725E-09
S9XA21	Uncharacterized protein	NA	47 ±7%	2.214E-14
T0MIA6	Gelsolin isoform 32	Binding protein	42 ±14%	1.427E-08
S9Z0C4	Pepsinogen 5, group I (Pepsinogen A)-like protein	Enzyme	42 ±8%	0
T0MKQ0	Complement component C7	Immune response	41 ±14%	6.833E-08
S9YTS3	Actin, cytoplasmic 1	Binding protein	35 ±7%	0
S9Y4T1	Xanthine dehydrogenase/oxidase	Enzyme	34 ±5%	0
S9Z0L8	Amine oxidase	Enzyme	15 ±4%	0
S9X2V0	UDP-Gal:βGlcNAc β 1,4-galactosyltransferase 1, membrane-bound form-like protein	Enzyme	14 ±7%	0
K7DXB9	α_{s1}-Casein	Cellular component	11 ±19%	0

SR	S9X3V4	TAF domain-containing protein	Binding protein	305 ±10%	0
	L0P3Z7	κ-Casein	Protein stabilization	207 ±2%	0
	T0MED9	Uncharacterized protein	Cellular component	189 ±5%	0
	P15522	Glycosylation-dependent cell adhesion molecule 1	Cellular component	176 ±7%	3.95E-15
	T0MGY4	Uncharacterized protein	NA	167 ±8%	4.272E-09
	S9X1L5	Lipoprotein lipase isoform 3	Enzyme	164 ±5%	0
	S9XC74	Osteopontin	Cell adhesion	138 ±5%	4.513E-10
	P15522-2	Glycosylation-dependent cell adhesion molecule 1	Cellular component	135 ±3%	0
	T0M5F7	Uncharacterized protein	NA	134 ±5%	4.272E-09
	S9X4G0	Neutrophil gelatinase-associated lipocalin-like protein	Binding protein	133 ±3%	0
	O97944	α_{s2}-Casein	Cellular component	126 ±3%	1.205E-11
	S9XGH7	Polyubiquitin-B	Binding protein	121 ±3%	2.282E-11
	T0M664	Zinc-α-2-glycoprotein-like protein	Binding protein	119 ±3%	9.939E-07
	M1E4K4	β-Casein	Cellular component	116 ±1%	0
	S9WPL9	Uncharacterized protein	Enzyme inhibitor	113 ±2%	3.526E-08
	S9YFK0	α-Lactalbumin	Enzyme	108 ±2%	1.765E-06
	S9WBY0	Clusterin	Binding protein	91 ±2%	2.651E-06
	S9Z0L8	Amine oxidase	Enzyme	89 ±2%	5.906E-07
	S9WF76	Lactadherin	Immune response	86 ±3%	8.764E-07
	S9WI87	Serum albumin	Cellular component	84 ±2%	0
	S9YD38	Complement factor B	Enzyme	83 ±3%	9.256E-09
	S9XI30	FABP domain-containing protein	Binding protein	83 ±3%	9.13E-08
	W6GH05	Lactotransferrin	Immune response	82 ±2%	0
	T0NN97	Uncharacterized protein	Enzyme inhibitor	81 ±2%	0
	S9YNY9	Nucleobindin-1	Binding protein	80 ±2%	0
	Q9GJW6	Peroxidase	Enzyme	72 ±3%	0
	S9WYL2	Serine carboxypeptidase CPVL	Enzyme	65 ±8%	7.266E-07
	S9X7Q1	Lactoperoxidase	Enzyme	63 ±5%	0
	S9Y2X0	Platelet glycoprotein 4	Immune response	47 ±5%	0
	K7DXB9	α_{s1}-Casein	Cellular component	47 ±11%	1.688E-11
	S9XEX5	Myelin proteolipid protein isoform 2	Cellular component	13 ±4%	1.42E-07
SSD	S9X3V4	TAF domain-containing protein	Binding protein	570 ±9%	0

	T0MED9	Uncharacterized protein	Cellular component	227 ±5%	0
	L0P3Z7	κ-Casein	Protein stabilization	208 ±2%	0
	T0MGY4	Uncharacterized protein	NA	187 ±8%	2.39E-13
	O97944	α_{s2}-Casein	Cellular component	170 ±3%	0
	S9X1L5	Lipoprotein lipase isoform 3	Enzyme	169 ±5%	0
	P15522-2	Glycosylation-dependent cell adhesion molecule 1	Cellular component	141 ±3%	0
	T0M664	Zinc-α-2-glycoprotein-like protein	Binding protein	139 ±3%	0
	S9X4G0	Neutrophil gelatinase-associated lipocalin-like protein	Binding protein	134 ±3%	0
	S9XC74	Osteopontin	Cell adhesion	131 ±5%	1.334E-08
	M1E4K4	β-Casein	Cellular component	123 ±1%	0
	S9W7K0	Uncharacterized protein	Immune response	121 ±3%	8.244E-08
	S9XE13	Uncharacterized protein	Cellular component	119 ±3%	7.017E-08
	S9Y4T1	Xanthine dehydrogenase/oxidase	Enzyme	111 ±2%	3.611E-08
	S9WPL9	Uncharacterized protein	Enzyme inhibitor	111 ±2%	2.66E-06
	S9YFK0	α-Lactalbumin	Enzyme	91 ±2%	5.864E-08
	S9XDK9	Complement C3	Enzyme inhibitor	90 ±2%	5.481E-08
	S9Z108	Plasma protease C1 inhibitor	Enzyme inhibitor	88 ±2%	7.355E-09
	S9WI87	Serum albumin	Cellular component	84 ±2%	0
	W6GH05	Lactotransferrin	Immune response	83 ±2%	0
	S9XCM5	Uncharacterized protein	Binding protein	81 ±3%	1.104E-13
	Q9GJW6	Peroxidase	Enzyme	78 ±3%	0
	S9X7Q1	Lactoperoxidase	Enzyme	68 ±4%	5.89E-14
	S9Y2X0	Platelet glycoprotein 4	Immune response	61 ±4%	0
	S9X0W6	Ig-like domain-containing protein	Immune response	18 ±12%	1.201E-09
SRO	S9X9X0	Vitelline membrane outer layer protein 1-like protein	Cellular component	373 ±16%	2.09E-12
	T0MII3	α-2-Macroglobulin-like protein	Enzyme inhibitor	331 ±4%	0
	S9X3V4	TAF domain-containing protein	Binding protein	257 ±12%	3.055E-10
	T0MED9	Uncharacterized protein	Cellular component	177 ±9%	6.018E-10
	S9XGH7	Polyubiquitin-B	Binding protein	167 ±8%	9.267E-08
	T0MGY4	Uncharacterized protein	NA	167 ±8%	2.29E-09

P15522	Glycosylation-dependent cell adhesion molecule 1	Cellular component	165 ± 10%	1.141E-06
S9XSQ6	Vitamin D-binding protein-like protein	Binding protein	132 ± 4%	4.739E-12
T0NN97	Uncharacterized protein	Enzyme inhibitor	125 ± 3%	2.909E-14
S9X4G0	Neutrophil gelatinase-associated lipocalin-like protein	Binding protein	121 ± 3%	8.183E-12
S9XYY2	Hemopexin	Binding protein	120 ± 3%	6.129E-08
S9WF76	Lactadherin	Immune response	115 ± 2%	7.552E-10
S9XCM5	Uncharacterized protein	Binding protein	78 ± 2%	0
S9YL21	Apolipoprotein A-I	Binding protein	72 ± 5%	8.374E-11
S9X7Q1	Lactoperoxidase	Enzyme	62 ± 8%	2.386E-08
S9Y4T1	Xanthine dehydrogenase/oxidase	Enzyme	61 ± 4%	0
S9XA21	Uncharacterized protein	NA	61 ± 8%	6.097E-08
S9Z0C4	Pepsinogen 5, group I (Pepsinogen A)-like protein	Enzyme	57 ± 7%	2.644E-14
W6GH05	Lactotransferrin	Immune response	56 ± 2%	0
S9XEX5	Myelin proteolipid protein isoform 2	Cellular component	56 ± 5%	0
S9YTS3	Actin, cytoplasmic 1	Binding protein	48 ± 10%	2.593E-12
T0MIA6	Gelsolin isoform 32	Binding protein	40 ± 18%	1.207E-06
S9YCI6	Peptidyl-prolyl cis-trans isomerase	Enzyme	38 ± 14%	3.501E-10
S9Z0L8	Amine oxidase	Enzyme	25 ± 4%	0
S9X2V0	UDP-Gal:βGlcNAc β 1,4-galactosyltransferase 1, membrane-bound form-like protein	Enzyme	23 ± 6%	0
K7DXB9	α_{s1}-Casein	Cellular component	10 ± 21%	0

FR, freeze-dried raw milk powder; FSD, spray dewatering-concentrated / freeze-dried milk powder; FRO, reverse osmosis-concentrated / freeze-dried milk powder; SR, spray-dried raw milk powder; SSD, spray dewatering-concentrated / spray-dried milk powder; SRO, reverse osmosis-concentrated / spray-dried milk powder.
